# Copy Number Variation detection from 1000 Genomes project exon capture sequencing data

**DOI:** 10.1186/1471-2105-13-305

**Published:** 2012-11-17

**Authors:** Jiantao Wu, Krzysztof R Grzeda, Chip Stewart, Fabian Grubert, Alexander E Urban, Michael P Snyder, Gabor T Marth

**Affiliations:** 1 Boston College, Boston, Chestnut Hill, MA, USA; 2 Stanford University School of Medicine, Stanford, CA, USA

## Abstract

**Background:**

DNA capture technologies combined with high-throughput sequencing now enable cost-effective, deep-coverage, targeted sequencing of complete exomes. This is well suited for SNP discovery and genotyping. However there has been little attention devoted to Copy Number Variation (CNV) detection from exome capture datasets despite the potentially high impact of CNVs in exonic regions on protein function.

**Results:**

As members of the 1000 Genomes Project analysis effort, we investigated 697 samples in which 931 genes were targeted and sampled with 454 or Illumina paired-end sequencing. We developed a rigorous Bayesian method to detect CNVs in the genes, based on read depth within target regions. Despite substantial variability in read coverage across samples and targeted exons, we were able to identify 107 heterozygous deletions in the dataset. The experimentally determined false discovery rate (FDR) of the cleanest dataset from the Wellcome Trust Sanger Institute is 12.5%. We were able to substantially improve the FDR in a subset of gene deletion candidates that were adjacent to another gene deletion call (17 calls). The estimated sensitivity of our call-set was 45%.

**Conclusions:**

This study demonstrates that exonic sequencing datasets, collected both in population based and medical sequencing projects, will be a useful substrate for detecting genic CNV events, particularly deletions. Based on the number of events we found and the sensitivity of the methods in the present dataset, we estimate on average 16 genic heterozygous deletions per individual genome. Our power analysis informs ongoing and future projects about sequencing depth and uniformity of read coverage required for efficient detection.

## Background

Copy Number Variations (CNVs) i.e. deletions and amplifications, are an essential part of normal human variability [1]. Specific CNV events have also been linked to various human diseases [2], including cancer [3,4] autism [5,6] and schizophrenia [7]. Historically, large CNV events can be observed using FISH [8] but systematic, genome-wide discovery of CNVs started with microarray-based methods [9-11] which can detect events down to 1 kb resolution. As with all hybridization based approaches, these methods are blind in repetitive and low complexity regions of the genome where probes cannot be designed. High throughput sequencing with next-generation technologies have enabled CNV detection at higher resolution (i.e. down to smaller event size), in whole-genome shotgun datasets [12-14]. However, despite decreasing costs, deep-coverage (≥ 25×) whole-genome data is still prohibitively expensive for routine sequencing of hundreds of samples, and in low-coverage (2-6× base coverage) datasets detection sensitivity and resolution is limited to long genomic events [1].

Targeted DNA capture technologies combined with high-throughput sequencing now provide a reasonable balance between coverage and sequencing cost in a substantial portion of the genome, and full-exome sequencing projects are presently collecting ≥ 25× average sequence coverage in thousands of samples. CNV events in exonic regions are important because the deletions of one or both copies, or amplifications affecting exons, are likely to incur phenotypic consequences.

Current algorithms for detecting CNVs in whole-genome shotgun sequencing data use one of four signals as evidence for an event: (1) aberrantly mapped mate-pair reads (RP or read pair methods); (2) split-read mapping positions (SR); (3) *de novo* assembly (AS); and (4) a significant drop or increase of mapped read depth (RD methods). Unfortunately, these methods are not generally applicable for CNV detection in capture sequence data without substantial modifications. SR, RP, and AS based methods are sensitive only to CNVs in which mapped reads or fragments span the event breakpoint (s). In the case of exon capturing data, this restricts detection to CNV events where at least one breakpoint falls in a targeted exon. RD based methods suffer from large fluctuations of sequence coverage stemming from variability in probe-specific hybridization affinities across different capture targets (in this case: exons) and sets of such targets (in our case: genes), and from the over-dispersion of the read coverage distribution in the same target across different samples. Presumably because of the technical challenges, and despite the importance of deletion or amplification events within exons, there are currently no reported CNV detection algorithms for targeted DNA capture based exon-sequencing data (with the exception of methods for tumor-normal datasets [15] where the read depth measured in the normal sample can be used for normalization – signal not available in the case of population sequencing).

In this study, we set out to develop a CNV detection algorithm for capture sequencing data. This algorithm is based on RD measurement, and detects samples with non-normal copy number in the capture target regions. As participants of the 1000 Genomes Project, we took part in the data analysis of the “Exon Sequencing Pilot” dataset [16], where 12,475 exons from over 900 genes were targeted and sequenced with a variety of DNA capture and sequencing technologies.

## Results and discussion

### Brief algorithmic overview

**Figure 1:**
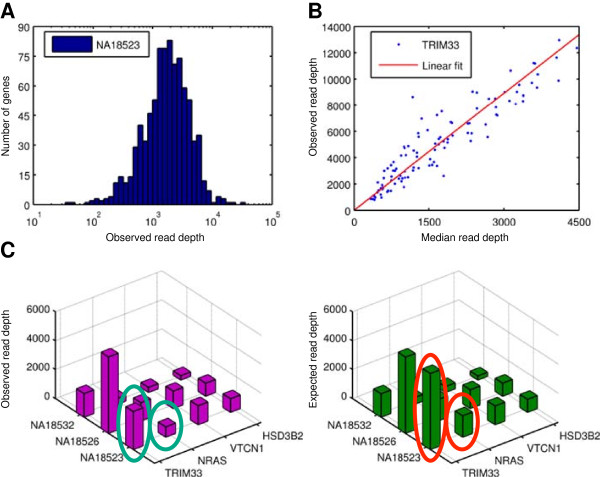
**Workflow of the CNV detection method. A**. Median Read Depth (MRD) is calculated for each sample, as a measure of sample coverage (NA18523 shown). **B**. The gene affinity is estimated for each gene as the slope of the least-square-error linear fit between MRD and RD for that gene (TRIM33 shown). **C**. Example of observed (magenta) and expected (green) read depth for three samples and four genes. The observed read depths were roughly half of the expected values for genes TRIM33 and NRAS, in sample NA18523, and detected as deletions.

Our algorithm is an extended version of RD-based CNV detection that aims to mitigate the vast target-to-target (and consequently gene-to-gene) heterogeneity of read coverage by normalization procedures roughly corresponding to those employed in CNV detection methods from microarray hybridization intensity data. The overall workflow of our method is shown in Figure 1 and described in greater detail in the Methods section. For a given gene in a given sample (we will use the abbreviation GSS: Gene-Sample Site throughout the paper), we define the read depth as the number of uniquely mapped reads whose 5’ end falls within any of the targeted exons within that gene. We compare this measurement with an expected read depth (Eq. 2, Methods), based on a “gene affinity” calculated from measured read depth for that gene across all samples (to account for across-target read coverage variance due to target-specific hybridization), and the overall read depth for the sample (to account for the variance of read coverage due to the overall sequence quantity collected for the sample under examination). We then use a Bayesian scheme to determine whether the measured coverage is consistent with normal copy number (e.g. CN = 2 for autosomes), or aberrant copy number (i.e. homozygous deletion: CN = 0, heterozygous deletion: CN = 1, or amplification: CN > 2). We have included two algorithmic variants: One is suitable for CNV events that occur at a low allele frequency (i.e. in a small fraction of the samples), and the other for capturing higher-frequency deletion events (see Methods).

### Dataset

**Figure 2:**
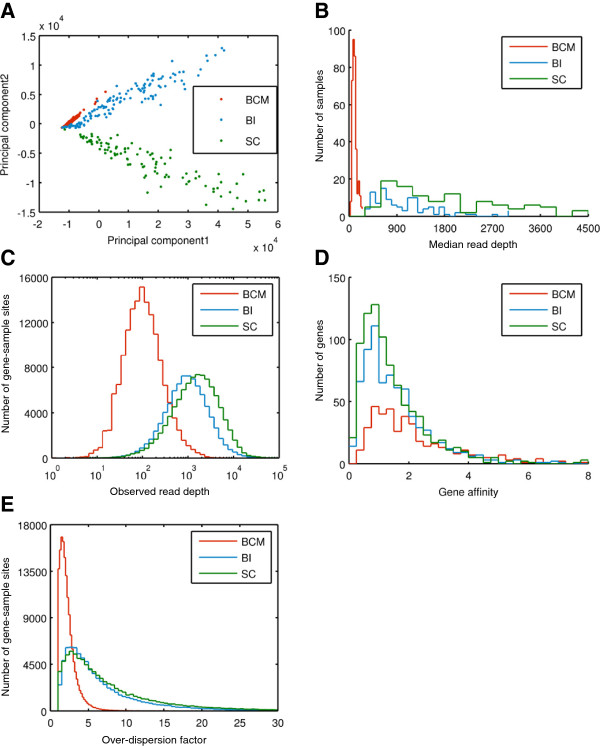
**Data characteristics for the 1000 genomes exon sequencing pilot datasets. A**. Principal component analysis of a “mixed” read depth matrix built with data from 3 different sequencing centers, SC (Wellcome Trust Sanger Institute), BI (Broad Institute) and BCM (Baylor College of Medicine). Each sample is represented as a point in the plot, with the first principal component plotted vs. the second principal component. Samples from different sequencing centers cluster separately from each other within this space, suggesting significant differences in the gene affinities among these three datasets. **B**. Distributions of MRD for each of the BCM, BI and SC samples **C**. Histogram of RD across all GSSs in the three datasets. **D**. Histogram of gene affinities across genes within each of the three datasets. **E**. Distributions of the RD over-dispersion factor (ODF) in our data.

In this study we analyzed the exon capture sequencing dataset collected by the 1000 Genomes Project Exon Sequencing Pilot, including 931 genes processed with Agilent liquid-phase and Nimblegen solid-phase capture methods, and sequenced from 697 individuals with Illumina paired-end and/or 454 technologies. The samples in the dataset have been sequenced by four different data collection centers (Washington University, WU; Wellcome Trust Sanger Institute, SC; Broad Institute, BI; and Baylor College of Medicine, BCM) using different pairings of capture and sequencing technologies (Table 1 and Table 2). As our method relies on an estimate of the gene-specific hybridization affinity, it requires that such affinities are consistent across all samples analyzed simultaneously. According to Principal Component Analysis of the observed read depths, (Figure 2A, see Methods), target and genes affinities are inconsistent across data from different centers, and therefore we analyzed each dataset separately. We only considered datasets with at least 100 samples (SC, BI, BCM) so we can obtain sufficient sample statistics across genes. After filtering out genes and samples that didn’t meet our minimum read depth requirements (see Methods), we were left with the following datasets: SC (862 genes in 106 individuals sequenced with Illumina), BI (739 genes in 110 samples sequenced with Illumina), and BCM (439 genes in 349 samples sequenced with 454) (Table 1). The number of genes that passed our filters was substantially lower in the BCM dataset both due to lower overall 454 coverage (see below), and because the longer 454 reads result in lower RD (fewer reads) when compared to shorter Illumina reads, even at equivalent base coverage.

**Table 1:** Properties of datasets from different sequencing centers

	**SC**	**BCM**	**BI**	**WU**
**Total sample count**	117	352	161	93
**Sample count after quality control**	106	349	110	82
**Technology**	Illumina	454	Illumina	Illumina
**Duplicate rate**	0.21	0.30	0.50	0.72
**Mapping quality (mean)**	50	33	45	51
**Base coverage(mean ± standard deviation)**	56 ± 34	23 ± 12	70 ± 61	29 ± 9
**Read depth per gene(mean ± standard deviation)**	2309 ± 3166	106 ± 171	1329 ± 2053	977 ± 1382
**MRD(mean ± standard deviation)**	1710 ± 1073	97 ± 52	1070 ± 803	599 ± 164
**Number of exons**	8174	8174	8174	8174
**Exons overlapped with segmental duplication regions**	458 (5.6%)	458 (5.6%)	458 (5.6%)	458 (5.6%)
**Number of genes (passing QC)**	862	439	739	1
**Genes overlapped with segmental duplication regions**	29 (3.3%)	11(2.5%)	23(3.1%)	0(0.0%)
**Over-dispersion factor(mean ± standard deviation)**	7.9 ± 8.2	2.1 ± 1.1	6.4 ± 5.5	N/A
**Quality index(mean ± standard deviation)**	9.4 ± 8.8	5.5 ± 2.3	7.6 ± 5.6	N/A
**Expected detection sensitivity based on quality index**	0.46	0.20	0.41	N/A
**Number of calls*h *= 0.65 either with or without a neighboring call**	36	4	56	N/A
**Number of calls*h *= 0.1 either with a neighboring call**	17	0	11	N/A

**Table 2:** Data characteristized by sequencing center and population

	**SC**						
	**CEU**	**CHB**		**JPT**		**TSI**	**YRI**
**Number of samples**	18	14		9		51	14
**Male/Female**	9/9	5/9		5/4		24/27	2/12
**Average read depth per gene**	1679	1701		1597		1617	1865
**Read length**	36	36		36		36	36
	**BCM**						
	**CEU**	**CHB**	**CHD**	**JPT**	**LWK**		**YRI**
**Number of samples**	40	62	78	16	108		45
**Male/Female**	20/20	15/47	38/40	5/11	51/57		22/23
**Average read depth per gene**	178	131	171	243	128		165
**Read length**	258	323	339	300	336		322
	**BI**						
	**CEU**	**CHB**	**CHD**	**JPT**			**YRI**
**Number of samples**	16	13	28	34			19
**Male/Female**	9/7	11/2	12/16	16/18			12/7
**Average read depth per gene**	1623	1631	1675	1104			1612
**Read length**	73	75	74	75			76

Population abbreviations:

CEU – Utah residents with Northern and Western European ancestry.

CHB – Han Chinese in Beijing.

CHD – Chinese in Denver, Colorado.

JPT – Japanese in Tokyo, Japan.

LWK – Luhya in Webuye, Kenya.

TSI – Toscans in Italy.

YRI – Yoruba in Ibadan, Nigeria.

### Sample coverage and gene affinities

As a metric of coverage for each sample, we calculated the sample-specific median gene RD, referred to as “Median Read Depth” (MRD); see Figure 1A and Methods. MRD was highest for the SC samples (1,710 ± 1,073, median 1,491 reads/gene; data presented as mean±standard deviation), see Figure 2B. MRD was somewhat lower for the BI samples (1,070 ± 803, median 860 reads/gene), and much lower in the BCM dataset (97 ± 52, median 87 reads/gene). As mentioned above, RD (distributed as in Figure 2C) is not determined by base coverage alone. Base coverage was highest in the BI data (70 ± 61, median 56 reads/base), followed by SC (56 ± 34, median 50 reads/base). The much lower RD in the 454 reads from BCM corresponds to only somewhat lower base coverage (23 ± 12, median 21 reads/base).

For each target we define a quantity, the “target affinity”, intended to describe the number of reads (RD) being mapped to a given target, relative to the sample-specific MRD over all capture targets. Analogously, we define the gene-specific affinity as the ratio of the number of reads (RD) mapped to the targets (exons) belonging to that gene and the gene-specific MRD for that same sample (see Methods, Figure 2D). In general, tighter distributions of affinities, with mean and median as close to 1 as possible, are desirable because these correspond to more even target coverage. The observed gene affinities for our datasets (Figure 2D) were as follows: SC (1.40 ± 1.43, median 1.04), BI (1.58 ± 1.59, median 1.20), and BCM (2.63 ± 3.03, median 1.73). Because of the more favorable gene affinities, we used the SC data as our primary dataset for method development and experimental validations.

### CNV candidates detected in the data

According to our Bayesian detection scheme, we call a heterozygous deletion event in a gene if the posterior probability value of CN = 1, i.e. P(CN=1 | RD) ≥ *h* where *h* is a pre-defined probability cutoff value. Similarly, a homozygous deletion is where P(CN=0 | RD) ≥ *h*. Although we detected both deletions and amplifications in the analyzed datasets, deletion events (even when in a heterozygous state) provide easier detectable signal than amplifications. For this reason we only discuss deletion events here and report candidate amplifications in Table 3.

**Table 3:** Gene duplication calls in the SC dataset

**Population**	**Sample**	**Gene name**	**Gene function**	**Chr**	**Start [bp]**	**End [bp]**	**Posterior probablity**	***RD***_**observed**_	***RD***_**expected**_
CEU	NA12348	CD300LB	CD300 molecule-like family member b	17	70030472	70039195	1.000	638	420
TSI	NA20533	CLDN10	claudin 10	13	95003009	95028269	1.000	2108	1582
CHB	NA18526	SNRNP27	small nuclear ribonucleoprotein 27 kDa (U4/U6.U5)	2	69974621	69977184	1.000	530	383
CHB	NA18532	CES1	carboxylesterase 1 (monocyte/macrophage serine esterase 1)	16	54401930	54424468	1.000	501	337
TSI	NA20752	NOM1	nucleolar protein with MIF4G domain 1	7	156435193	156455158	1.000	1335	966
TSI	NA20796	AHNAK	AHNAK nucleoprotein	11	62040792	62059238	1.000	7330	5169
TSI	NA20796	ZNF264	zinc finger protein 264	19	62408577	62416161	0.999	1276	888
TSI	NA20801	GPR128	G protein-coupled receptor 128	3	101811391	101896535	0.998	14747	8265
TSI	NA20772	STX16	syntaxin 16	20	56660469	56684753	0.998	2101	1605
TSI	NA20769	MRPS6	mitochondrial ribosomal protein S6	21	34419511	34436770	0.998	1585	1203
TSI	NA20774	ELAVL4	ELAV (embryonic lethal, abnormal vision, Drosophila)-like 4 (Hu antigen D)	1	50383216	50439437	0.998	782	567
TSI	NA20804	CYP2A13	cytochrome P450, family 2, subfamily A, polypeptide 13	19	46291375	46293686	0.997	1289	984
TSI	NA20774	CREB5	cAMP responsive element binding protein 5	7	28494318	28825421	0.996	1435	954
TSI	NA20796	ZNF32	zinc finger protein 32	10	43459504	43461587	0.996	911	646
TSI	NA20520	C6orf145	chromosome 6 open reading frame 145	6	3668852	3683381	0.995	2015	1601
CEU	NA12348	GDNF	glial cell derived neurotrophic factor	5	37851510	37870647	0.994	306	217
CHB	NA18561	PSMB4	proteasome (prosome, macropain) subunit, beta type, 4	1	149638688	149640730	0.986	3461	2216
CEU	NA12546	DAZAP2	DAZ associated protein 2	12	49920394	49922509	0.985	2265	1651
TSI	NA20752	AATF	apoptosis antagonizing transcription factor	17	32380539	32488077	0.976	1157	843
CEU	NA12749	PAQR5	progestin and adipoQ receptor family member V	15	67439474	67483215	0.976	1684	1239
TSI	NA20769	BCL2L11	BCL2-like 11 (apoptosis facilitator)	2	111597794	111638279	0.965	1813	1435
TSI	NA20804	PILRA	paired immunoglobin-like type 2 receptor alpha	7	99809603	99835466	0.909	962	752
TSI	NA20589	C8orf85	chromosome 8 open reading frame 85	8	118019664	118024121	0.903	147	91
TSI	NA20752	CCKAR	cholecystokinin A receptor	4	26092358	26100987	0.902	712	532
JPT	NA18973	HBG2	hemoglobin, gamma G	11	5278820	5523329	0.901	4151	3094
TSI	NA20774	HIPK1	homeodomain	1	114298778	114317657	0.900	2374	1626
TSI	NA20774	ODC1	ornithine decarboxylase 1	2	10498301	10502609	0.897	1489	935
TSI	NA20796	STBD1	starch binding domain 1	4	77446947	77450177	0.885	978	664
TSI	NA20589	CRIPAK	cysteine-rich PAK1 inhibitor	4	1378300	1379640	0.877	76	38
YRI	NA19189	PSMB4	proteasome (prosome, macropain) subunit, beta type, 4	1	149638688	149640730	0.853	2622	2090
TSI	NA20774	STX16	syntaxin 16	20	56660469	56684753	0.811	949	704
JPT	NA18980	CES1	carboxylesterase 1 (monocyte/macrophage serine esterase 1)	16	54401930	54424468	0.788	1679	1036
TSI	NA20774	PAQR5	progestin and adipoQ receptor family member V	15	67439474	67483215	0.788	1048	676
CHB	NA18561	CRNN	cornulin	1	150648694	150651333	0.778	4845	3172
TSI	NA20774	DKK4	dickkopf homolog 4 (Xenopus laevis)	8	42350775	42353720	0.760	493	362
TSI	NA20589	NOM1	nucleolar protein with MIF4G domain 1	7	156435193	156455158	0.740	1052	801
TSI	NA20769	RNF122	ring finger protein 122	8	33525813	33535831	0.734	2574	2004
TSI	NA20796	ZNF521	zinc finger protein 521	18	20896674	21184908	0.721	3536	2738
TSI	NA20769	VLDLR	very low density lipoprotein receptor	9	2625453	2631499	0.676	2092	1624

**Figure 3:**
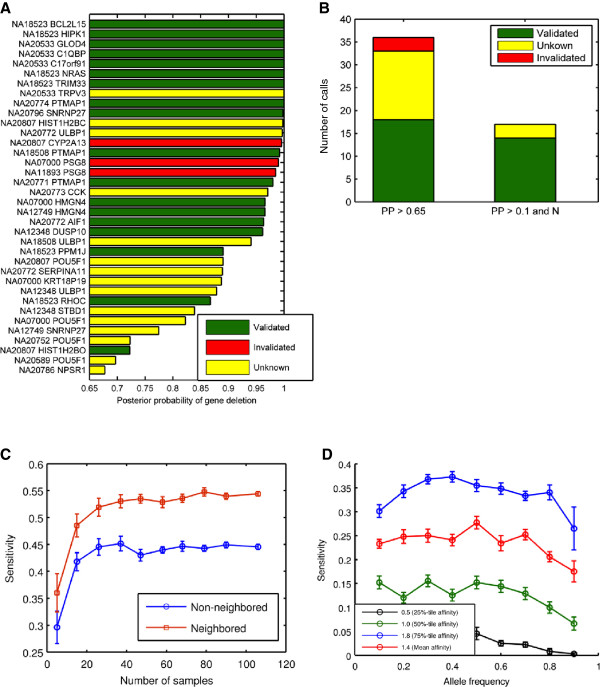
**Detected CNV events. A**. Top-ranked (by posterior probability) deletion events in the SC dataset. **B**. Validation results for different callsets (left – without neighboring information, right – with use of neighboring information). Green denotes events positively validated either in our experiments or as known events [17]; red – calls validated negatively in our experiments; yellow – calls without validation status (not submitted for validation or validation experiments without conclusive outcomes). **C**. Detection sensitivity as a function of number of samples. **D.** Sensitivity of detecting common CNV as a function of the deleted allele frequency.

Using a cutoff value *h *= 0.65, we detected deletion 96 events in the three datasets (36 in SC, 56 in BI, and 4 in BCM), all heterozygous deletions (Table 4, Table 5 and Table 6). The top ranked deletions are shown in Figure 3A. Most of the events were found in the Tuscan population, which constituted about half of the sample set. 10 of 36 gene deletions in the SC dataset were found in two samples (NA18523 and NA20533), clustered in a contiguous string of deleted genes extending approximately 3 Mb on chromosome 1 and 17, respectively, a genomic deletion event that we were also able to find in the 1000 Genomes Project whole-genome Low Coverage Pilot data [16] from the same samples (data not shown).

**Table 4:** Gene deletion calls in the SC dataset

**Population**	**Sample**	**Gene name**	**Gene function**	**Chr**	**Start [bp]**	**End [bp]**	**Posterior probability**	***RD***_**observed**_	***RD***_**expected**_
YRI	NA18523	BCL2L15	BCL2-like 15	1	114225268	114231520	1.000	533	1158
YRI	NA18523	HIPK1	homeodomain interacting protein kinase 1	1	114298778	114317657	1.000	2539	5272
TSI	NA20533	GLOD4	glyoxalase domain containing 4	17	610163	632245	1.000	1322	2295
TSI	NA20533	C1QBP	complement component 1, q subcomponent binding protein	17	5277059	5282317	1.000	793	1416
TSI	NA20533	C17orf91	chromosome 17 open reading frame 91	17	1562414	1563890	1.000	369	574
YRI	NA18523	NRAS	neuroblastoma RAS viral (v-ras) oncogene homolog	1	115052679	115060304	1.000	702	1462
YRI	NA18523	TRI3	tripartite motif-containing 33	1	114741793	114808533	1.000	2610	5225
TSI	NA20533	TRPV3	transient receptor potential cation channel, subfamily V, member 3	17	3363961	3404894	1.000	3365	5275
TSI	NA20774	PTMAP1	prothymosin, alpha pseudogene 1 (gene sequence 26)	6	30725671	30728671	1.000	132	260
TSI	NA20796	SNRNP27	small nuclear ribonucleoprotein 27 kDa (U4/U6.U5)	2	69974621	69977184	0.998	105	194
TSI	NA20807	HIST1H2BC	histone cluster 1, H2bc	6	26231731	26232111	0.998	42	90
TSI	NA20772	ULBP1	UL16 binding protein 1	6	150331436	150332954	0.997	104	205
TSI	NA20807	CYP2A13	cytochrome P450, family 2, subfamily A, polypeptide 13	19	46291375	46293686	0.996	126	204
YRI	NA18508	PTMAP1	prothymosin, alpha pseudogene 1 (gene sequence 26)	6	30725671	30728671	0.992	145	230
CEU	NA07000	PSG8	pregnancy specific beta-1-glycoprotein 8	19	47950287	47960273	0.990	29	70
CEU	NA11893	PSG8	pregnancy specific beta-1-glycoprotein 8	19	47950287	47960273	0.985	43	86
TSI	NA20771	PTMAP1	prothymosin, alpha pseudogene 1 (gene sequence 26)	6	30725671	30728671	0.980	533	862
TSI	NA20773	CCK	cholecystokinin	3	42274594	42280126	0.971	282	474
CEU	NA07000	HMGN4	high mobility group nucleosomal binding domain 4	6	26653414	26653686	0.966	68	132
CEU	NA12749	HMGN4	high mobility group nucleosomal binding domain 4	6	26653414	26653686	0.966	156	286
TSI	NA20772	AIF1	allograft inflammatory factor 1	6	31692086	31692262	0.964	51	124
CEU	NA12348	DUSP10	dual specificity phosphatase 10	1	219942377	219946216	0.962	155	242
YRI	NA18508	ULBP1	UL16 binding protein 1	6	150331436	150332954	0.941	40	79
YRI	NA18523	PPM1J	protein phosphatase, Mg2+/Mn2+ dependent, 1 J	1	113056116	113057756	0.891	560	924
TSI	NA20807	POU5F1	POU class 5 homeobox 1	6	31240884	31241803	0.891	124	193
TSI	NA20772	SERPINA11	serpin peptidase inhibitor, clade A (alpha-1 antiproteinase, antitrypsin), member 11	14	93978696	93984864	0.889	786	1243
CEU	NA07000	KRT18P19	keratin 18 pseudogene 19	12	51630379	51632393	0.887	85	174
CEU	NA12348	ULBP1	UL16 binding protein 1	6	150331436	150332954	0.879	49	88
YRI	NA18523	RHOC	ras homolog gene family, member C	1	113054308	113055529	0.867	557	955
CEU	NA12348	STBD1	starch binding domain 1	4	77446947	77450177	0.839	246	395
CEU	NA07000	POU5F1	POU class 5 homeobox 1	6	31240884	31241803	0.823	106	169
CEU	NA12749	SNRNP27	small nuclear ribonucleoprotein 27 kDa (U4/U6.U5)	2	69974621	69977184	0.775	142	216
TSI	NA20752	POU5F1	POU class 5 homeobox 1	6	31240884	31241803	0.723	76	142
TSI	NA20807	HIST1H2BO	histone cluster 1, H2bo	6	27969220	27969600	0.723	48	88
TSI	NA20589	POU5F1	POU class 5 homeobox 1	6	31240884	31241803	0.697	61	117
TSI	NA20786	NPSR1	neuropeptide S receptor 1	7	34884213	34884321	0.678	51	88

**Table 5:** Gene deletion calls in the BI dataset

**Population**	**Sample**	**Gene name**	**Gene function**	**Chr**	**Start [bp]**	**End [bp]**	**Posterior probability**	***RD***_**observed**_	***RD***_**expected**_
CHD	NA18695	TPM3	tropomyosin 3	1	152396739	152422219	1.000	166	337
JPT	NA19066	TPM3	tropomyosin 3	1	152396739	152422219	1.000	169	288
CHD	NA18687	RPL27A	ribosomal protein L27a	11	8661325	8663929	1.000	93	182
JPT	NA18983	POU5F1	POU class 5 homeobox 1	6	31240357	31241803	1.000	122	256
JPT	NA19066	POU5F1	POU class 5 homeobox 1	6	31240357	31241803	1.000	166	318
JPT	NA19066	RPL27A	ribosomal protein L27a	11	8661325	8663929	1.000	106	203
CHD	NA18687	TPM3	tropomyosin 3	1	152396739	152422219	1.000	155	258
CHD	NA18687	POU5F1	POU class 5 homeobox 1	6	31240357	31241803	1.000	156	285
JPT	NA19054	TPM3	tropomyosin 3	1	152396739	152422219	1.000	135	230
CHD	NA18695	POU5F1	POU class 5 homeobox 1	6	31240357	31241803	1.000	194	371
JPT	NA18960	SETD8	SET domain containing (lysine methyltransferase) 8	12	122441130	122455574	1.000	221	347
CHD	NA18164	RPL27A	ribosomal protein L27a	11	8661325	8663929	1.000	129	223
JPT	NA19054	POU5F1	POU class 5 homeobox 1	6	31240357	31241803	1.000	130	254
CHD	NA18695	SETD8	SET domain containing (lysine methyltransferase) 8	12	122441130	122455574	1.000	142	309
CHD	NA18695	RPL27A	ribosomal protein L27a	11	8661325	8663929	1.000	128	238
CHD	NA18695	AKR1B1	aldo-keto reductase family 1, member B1 (aldose reductase)	7	133778020	133787045	1.000	310	554
CHD	NA18164	HAX1	HCLS1 associated protein X-1	1	152512874	152514801	1.000	214	339
CHD	NA18687	SETD8	SET domain containing (lysine methyltransferase) 8	12	122441130	122455574	1.000	125	237
JPT	NA19054	HFE	hemochromatosis	6	26201326	26202433	1.000	56	122
JPT	NA18983	RPL27A	ribosomal protein L27a	11	8661325	8663929	0.990	95	164
JPT	NA18983	TPM3	tropomyosin 3	1	152396739	152422219	0.990	147	232
JPT	NA19561	TRIM55	tripartite motif-containing 55	8	67202058	67209944	0.990	119	193
CHD	NA18687	RBMS1	RNA binding motif, single stranded interacting protein 1	2	160840394	160932124	0.990	334	575
CHB	NA18757	CRIPAK	cysteine-rich PAK1 inhibitor	4	1378300	1379640	0.990	327	669
JPT	NA19054	PSAT1	phosphoserine aminotransferase 1	9	80109471	80113319	0.980	140	253
JPT	NA19066	PSAT1	phosphoserine aminotransferase 1	9	80109471	80113319	0.980	190	317
CHD	NA18164	TPM3	tropomyosin 3	1	152396739	152422219	0.980	209	317
JPT	NA19568	OR8A1	olfactory receptor, family 8, subfamily A, member 1	11	123945175	123946141	0.980	471	764
JPT	NA19066	RAN	RAN, member RAS oncogene family	12	129923334	129926424	0.980	229	462
CHD	NA18695	KLHL12	kelch-like 12	1	201128284	201160913	0.970	767	1358
JPT	NA19066	SETD8	SET domain containing (lysine methyltransferase) 8	12	122441130	122455574	0.970	154	265
JPT	NA19066	RPS15A	ribosomal protein S15a	16	18706886	18707936	0.960	83	161
CHD	NA18695	RPS15A	ribosomal protein S15a	16	18706886	18707936	0.960	88	188
CHD	NA18687	KLHL12	kelch-like 12	1	201128284	201160913	0.960	621	1041
JPT	NA18983	SETD8	SET domain containing (lysine methyltransferase) 8	12	122441130	122455574	0.960	120	213
JPT	NA18983	DCTN5	dynactin 5 (p25)	16	23560365	23585966	0.960	177	298
JPT	NA18983	EIF2B5	eukaryotic translation initiation factor 2B, subunit 5 epsilon, 82 kDa	3	185500333	185509372	0.940	856	1482
CHD	NA18687	ARG2	arginase, type II	14	67187855	67187951	0.940	28	62
CHD	NA18695	PSAT1	phosphoserine aminotransferase 1	9	80109471	80113319	0.930	221	371
CHD	NA18695	RBMS1	RNA binding motif, single stranded interacting protein 1	2	160840394	160932124	0.900	442	750
JPT	NA19561	OR8A1	olfactory receptor, family 8, subfamily A, member 1	11	123945175	123946141	0.890	254	466
YRI	NA19247	TIMM8B	translocase of inner mitochondrial membrane 8 homolog B (yeast)	11	111461229	111462657	0.880	40	89
CHD	NA18164	POU5F1	POU class 5 homeobox 1	6	31240357	31241803	0.850	226	349
CHD	NA18164	KLHL12	kelch-like 12 (Drosophila)	1	201128284	201160913	0.800	803	1276
CHD	NA18164	SETD8	SET domain containing (lysine methyltransferase) 8	12	122441130	122455574	0.790	181	291
CHD	NA18687	RPS15A	ribosomal protein S15a	16	18706886	18707936	0.790	81	144
JPT	NA19066	EIF2B5	eukaryotic translation initiation factor 2B, subunit 5 epsilon, 82 kDa	3	185500333	185509372	0.780	1137	1840
JPT	NA19568	GABARAPL2	GABA(A) receptor-associated protein-like 2	1	157676173	157676631	0.760	254	476
JPT	NA19560	OR8A1	olfactory receptor, family 8, subfamily A, member 1	11	123945175	123946141	0.750	614	1119
JPT	NA19058	RPL27	ribosomal protein L27	17	38404294	38408463	0.730	356	518
CHD	NA18699	SDPR	serum deprivation response	2	192408894	192419896	0.720	524	1033
JPT	NA18983	SPRR2G	small proline-rich protein 2 G	1	151388989	151389210	0.670	81	147
JPT	NA19066	SPRR2G	small proline-rich protein 2 G	1	151388989	151389210	0.670	105	182
JPT	NA19066	RBMS1	RNA binding motif, single stranded interacting protein 1	2	160840394	160932124	0.670	404	642
JPT	NA19054	EIF2B5	eukaryotic translation initiation factor 2B, subunit 5 epsilon, 82 kDa	3	185500333	185509372	0.670	869	1470
CHD	NA18695	RAN	RAN, member RAS oncogene family	12	129923334	129926424	0.660	290	539

**Table 6:** Gene deletion calls in the BCM dataset

**Population**	**Sample**	**Gene name**	**Gene function**	**Chr**	**Start [bp]**	**End [bp]**	**Posterior probability**	***RD***_**observed**_	***RD***_**expected**_
LWK	NA19355	MBD5	methyl-CpG binding domain protein 5	2	148932798	148986980	0.999	618	973
CHD	NA17970	MTERFD2	MTERF domain containing 2	2	241684086	241687982	0.996	255	393
CHB	NA18618	GABARAPL2	GABA(A) receptor-associated protein-like 2	16	74159436	74168768	0.800	58	99
CHD	NA18135	PSMB4	proteasome (prosome, macropain) subunit, beta type, 4	1	149638688	149640929	0.729	390	605

When two or more gene deletions are detected in close proximity, it is likely that these events are part of a single, longer genomic deletion spanning the genes. With this in mind, we searched the sequenced genes for deletion events at a lower probability cutoff value (*h *= 0.1), but required that an immediate neighbor of a candidate gene be located within 3 Mbp and also show evidence for a deletion at the same probability cutoff. This procedure produced 17 heterozygous deletion calls in the SC dataset, 11 calls in the BI dataset (but no such calls were made in the BCM dataset). The union of both callsets (i.e. those made with and without use of neighboring information) resulted in a total of 107 unique deletion events (41 in SC dataset, 62 in BI, and 4 in BCM).

We note that none of the events we detected in our data were at high allele frequency. In fact, even the most “common” events were only present in two samples, as heterozygotes.

### Call-set accuracy assessment

To assess the accuracy of deletion calls made in the SC dataset, we performed experimental validations on calls made with posterior probability ≥ 0.65 without neighbor information, using qPCR (see Methods). The validation results are summarized in Figure 3B. Of the 36 calls made, we evaluated 26. All 22 calls with posterior probability ≥ 0.95 and 4 out of 12 calls (randomly selected) with posterior probability between 0.65 and 0.95 were submitted for validation. 6 were considered positively validated as they appeared in an earlier publication [17] and 20 were validated *de novo* using qPCR. The qPCR validations produced positive results for 12 calls (measured fold change < 0.7) and negative results for 3 calls (measured fold change > 0.8). The validation results for the remaining 5 were inconclusive. All the 17 neighbored calls with posterior probability ≥ 0.1 were selected for validation. 7 were considered valid per previous publication [17], 7 were positively validated *de novo* and none was found invalid; validation was not obtained for the remaining 3. The union of those two callsets counted 41 calls and 32 of them were evaluated. Among these 32 calls 7 were considered positively validated per previous publication [17], 14 were positively validated *de novo*, 3 were invalidated, 5 were inconclusive and 3 did not obtain the validation results. The numbers of validated calls are presented in Table 7. The selection procedure for site validation was as follows: (1) We selected sites for validation (in some categories, all candidates, in others, a random selection); (2) we searched the literature, and removed from the validation list events that we found as validated in one of the publications we consulted; (3) events that remained on the list were sent for experimental validation. The overall FDR for the union of calls made with and without neighboring information can be estimated as 12.5% (3/24).

**Table 7:** Validation results

	**Posterior ≥ 0.95 without neighbor information**	**0.65 ≤ Posterior < 0.95 without neighbor information**	**Posterior ≥ 0.1 with neighbor information**
**Validated per previous publication**	4	2	7
**Validated positively de novo**	11	1	7
**Validated inconclusively de novo (intermediate fold change)**	4	1	0
**Validated negatively de novo**	3	0	0
**Submitted for validation but without result or no validation attempt**	0	10	3
**Total calls**	22	14	17

### Sensitivity

We performed simulations to assess the detection efficiency of our method, both for individual gene and for pairs of neighboring genes deletions. Specifically, in each sample we randomly selected (a) 5 out of 862 genes in one simulation and (b) 5 pairs of neighboring genes in another simulation. In the selected genes we down-sampled the actual read depth seen in the experimental data by a factor of 2 to simulate a heterozygous deletion. The results of those simulations are presented in Figure 3C. Of the 530 gene deletions, we detected 237 (45%). Of the 530 gene-pair deletions we detected 287 (54%). We also performed simulations on smaller subsets of the original 106 samples to assess the impact of sample size on detection sensitivity. Reduction of sample size did not substantially degrade detection sensitivity as long as the number of samples was >20. Therefore, our detection efficiency is 40-45% without using neighboring information and approximately 50-55% with the use of neighboring information, in the SC dataset.

In addition to simulations, we compared our dataset to a published study [17]. This study reported 12 heterozygous deletion events in samples and genes (in our terminology, GSS) that were part of our analyzed dataset. We detected 6 of these 12 events, which is broadly consistent with our overall sensitivity estimate.

Finally, we investigated our sensitivity to common events (see Methods) using simulations. Figure 3D shows detection sensitivity as a function of gene-level affinity: for a gene affinity value of 1.8 (representing the 75^th^ percentile of our data), sensitivity to common events (allele frequency between 10% and 90%) approaches 40%. Note that the detection efficiency starts to decrease at high allele frequency (> 70%) due to a reduction of the overall read depth because more samples have a deletion and a corresponding depleted read depth signal. We can also see that the median gene affinity is substantially lower than the mean because the distribution of gene affinity has a long tail at the high end (Figure 2D). Since sensitivity is directly related to the gene affinity, the simulated data with the substantially higher mean gene affinity (red) has better sensitivity than with the substantially lower median gene affinity (green).

### The number of CNV events in the samples

**Figure 4:**
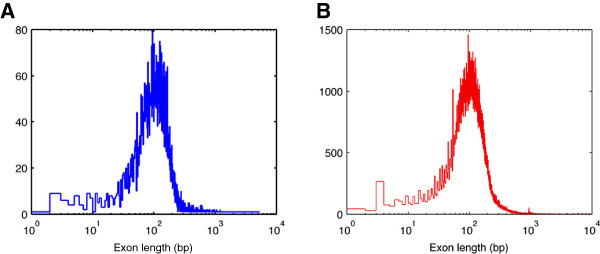
**Exon length distribution. A**. Exon length distribution in the gene list used for our analysis. (median: 125 bp, standard deviation: 236 bp) **B**. Exon length distribution of the whole exome. (median: 127 bp, standard deviation: 264 bp) These two distributions are very similar to each other, suggesting our estimation of the number of events per sample is unbiased and is representative for a whole-exome study.

We estimated the total number of gene deletions in the SC dataset from the number of detected events (41), the FDR (12.5%) and the detection efficiency (45%), as ~66, or a nominal 0.62 deletions per sample. By projecting the per-sample number, corresponding to 3.9% of the exome (862 genes of 21,999), onto the whole exome, our estimate for the average number of genic deletion events is 16 ± 4 per sample. This estimation is representative for the whole-exome sequencing data since the 1000 Genomes Exon Pilot Project randomly selected all the exon targets from the CCDS collection. Our gene set is therefore a quasi-random sampling of known human genes, with no intentional enrichment for any given gene family. Figure 4A and B show the distributions of exon length in the gene list used for our analysis and the full human exome. There is no significant difference between these two distributions: the median and the standard deviation of the exon length for our study are 125 bp and 236 bp, whereas the corresponding values for the whole exome are 127 bp and 264 bp. The similarity of these two distributions suggests that our estimation of the number of events per sample is unbiased and is representative for a whole-exome analysis.

### Detection efficiency as a function of data quantity and data quality

As discussed earlier, our algorithm’s sensitivity was ~45% at ~87.5% accuracy. Both sensitivity and accuracy are considerably lower than achievable for SNP detection in the same datasets [16]. This poses the more general question of how detection efficiency is influenced by sample size, data quantity, and data quality. Our simulations show that sensitivity only modestly depends on sample size, above approximately 20 samples (Figure 3C).

**Figure 5:**
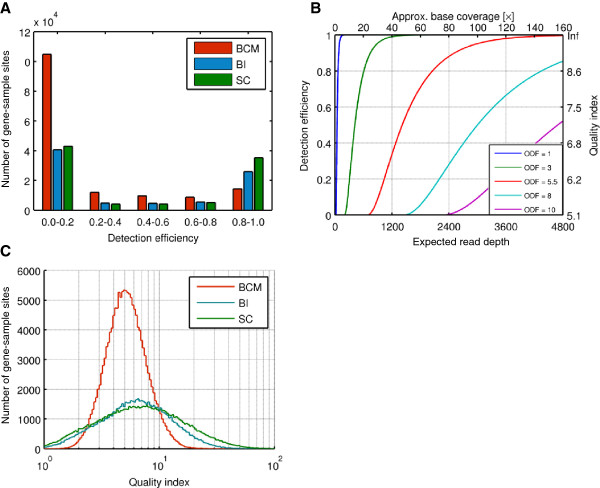
**Detection efficiency. A**. Distributions of the detection efficiency estimated from the quality index for each gene-sample site. **B**. Theoretical detection efficiency (at posterior probability cutoff *h *= 0.65) as a function of expected read depth, plotted for various values of the over-dispersion factor. **C**. Histograms of the quality index (QI) distribution in the three datasets. Overall, QI was highest in SC: 9.4±8.8 (median 6.6); second highest in BI: QI = 7.6 ± 5.6 (median 6.2); and lowest in BCM: QI = 5.5 ± 2.3 (median 5.0).

We found that the primary factors that determine detection efficiency are (i) sequence coverage, or more precisely, RD (higher RD supplies more statistical power to detect systematic changes in coverage); (ii) the level of over-dispersion of the RD distribution for individual genes (the more the RD distribution departs from an expected Poisson distribution, the less one can rely on the statistics); and (iii) the shape of the distribution of RD across all genes in the dataset, determined by the gene affinities (uneven distribution means that detection power is low in a high fraction of the genes, but this effect is not compensated by the extra coverage in other, “over-sequenced” genes where detection efficiency is already high, see Figure 5A below. Favorable scenarios therefore involve distributions in which all or most genes have sufficient RD for detection).

For each gene, we compute a quality index (QI) taking into account the variance of the expected read depth for that gene (assuming the ideal, Poisson distribution), *RD*_exp_, and a over-dispersion factor, ODF, that quantifies the over-dispersion of RD relative to the Poisson expectation:

(1) $$QI=\frac{\sqrt{RD_{\text{expected}}}}{ODF}$$

QI is directly related to detection sensitivity (see Additional file 1 for the exact formula and its derivation), as shown in Figure 5B. According to our power calculations, for the posterior detection threshold value we used in this study (*h *= 0.65), sensitivity is completely diminished for genes with QI < 5.1. QI ≥ 7.2 is required to achieve 50% sensitivity, and QI ≥ 9.5 to achieve 90% sensitivity. This estimated sensitivity from QI is made only for heterozygous deletions. To achieve the same sensitivity for detecting higher copy number variation (CN≥3), higher QI value will be required since the difference of prior probability between higher copy and normal copy (CN=2) is greater than that between heterozygous deletion and normal copy (Table 8).

**Table 8:** **Nominal prior probabilities corresponding to the range of gene region copy numbers derived from Conrad et al. **[[17]]

**Copy number**	**Prior probability per gene**
0	6.34·10^-4^
1	2.11·10^-3^
2	9.96·10^-1^
3	5.38·10^-4^
4	6.68·10^-4^
5	3.57·10^-5^
6	7.52·10^-6^
7	1.39·10^-6^
8	3.61·10^-7^
9	4.37·10^-8^

The distributions of QI values in our three datasets are shown in Figure 5C. Overall, QI was highest in SC: 9.4 ± 8.8 (median 6.6); second highest in BI: QI = 7.6 ± 5.6 (median 6.2); and lowest in BCM: QI = 5.5 ± 2.3 (median 5.0). The corresponding distributions of detection efficiency values are shown in Figure 5A. Because detection efficiency increases abruptly from 0 to almost 1 over a narrow range of QI values (note the mapping between the vertical axes in Figure 5B), the distribution of detection sensitivity (Figure 5A) is strongly bimodal, with the vast majority of GSS having either close to zero or close to 100% sensitivity. Even in the SC dataset with the highest overall QI values, in less than half of the GSS does the quantity and quality of the data support >80% detection efficiency. There was also very substantial variation across samples: only 15 of the 106 SC samples had sufficiently high coverage to support ≥ 90% overall sensitivity, and in 22 samples overall sensitivity was below 10%.

Given that QI improves only with the square root of RD, over-dispersion can profoundly influence detection performance, as shown in Figure 5B. The ODF values we chose for this figure correspond to the 25^th^, 50^th^ and 75^th^ percentile, and the mean values (ODF=3, 5.5, 10, and 8, respectively) in the SC dataset. Using the observed distribution of QI in the SC dataset, we predict ~46% sensitivity, in good agreement with our estimate based on simulations.

The QI formulation permits one to estimate CNV (or specifically in our case, heterozygous deletion) detection power in any given exon capture dataset, based on the read mappings. One can also use the formulation to calculate the amount of base coverage required for a given level of desired power, to guide data collection. For example, using the distributions of QI values in the SC dataset, one would need to collect an overall 110× coverage, assuming 36 bp reads, to achieve 60% detection power, and 320× coverage to achieve 80% detection power. However, if DNA capture methods improved to support a median ODF=3, assuming an accordingly scaled version of the observed distribution of QI in the SC dataset, one would only need to collect 33× coverage for 60% power, and 96× for 80% power. It is important to also point out that, in the case of whole-exome data, sensitivity would also improve just by virtue of the higher density of targeted genes, if one were to integrate in one’s pipeline neighbor-gene based detection.

### Methodology comparison with CoNIFER

Krumm and his colleagues recently published a method, CoNIFER [18], that also used read-depth signal to detect CNV in the exome capturing sequencing data. Like our method, CoNIFER normalizes the read depth signal in order to discover the CNV. However, it is quite different for these two algorithms in the approach of calling samples copy number variants on the basis that they present aberrant read depth. As we mentioned previously, our method deploys specific models for copy numbers 0, 1, 2, and is capable of detecting both rare, intermediate frequency, and common CNV events. On the other hand, CoNIFER deploys singular value decomposition (SVD) to remove noise from the read depth data, and interprets the first “k” singular values as noise in the data. This approach may identify systematic variance in the data caused by a high-frequency CNV event as noise and removes it. Therefore CoNIFER has limited power for detecting common CNV events. On the other hand, our method is capable of detecting CNV events on the entire frequency spectrum, and is therefore more generally applicable.

## Conclusions

We have developed a novel, Bayesian method to identify CNVs in exon-capture data. We applied this method (and a simple extension using neighbor-gene information) to the 1000 Genomes Project Exon Sequencing Pilot dataset. We were able to achieve reasonable sensitivity and specificity in a dataset that was optimized for SNP discovery and, as discussed above, is far from ideal for CNV detection. The main accomplishment of this work is that we provide a statistically rigorous algorithm for CNV detection in exon capture data, backed by experimental validations, that can be applied to the thousands of exomes sequenced to date in various medical projects, and to nascent and ongoing projects targeting increasingly higher numbers of samples. Our formulation allows investigators to assess detection power in existing datasets and to take into account CNV detection power during experimental design for future datasets. We also uncovered >100 heterozygous deletion events in the 1000 Genomes samples we examined, allowing us to estimate the average number of heterozygous deletions per exome (as ~16 events per exome). Because we focused on algorithm development functional assessment of these sites is beyond the scope of this study. Nevertheless, these and other gene deletions that will be found using our methods are very likely to uncover events with strong functional significance.

## Methods

The overall detection workflow (shown in Figure 1) consists of three main steps. (1) We tabulate the *observed* read depth for every GSS. (2) We determine whether the distribution of read depth for a specific gene distribute across samples should be modeled using simple uni-linear fit or using a more sophisticated tri-linear fit. (3) If the simple uni-linear fit is found suitable, we determine an *expected* read depth for every GSS under a null hypothesis of a normal copy number, using a simple linear fit model. (4) Subsequently, we compare the observed read depth for a GSS to the corresponding expectation and calculate a Bayesian posterior probability for each copy number considered (CN=0-9), threshold these, and report events with a non-normal CN. (5) If data do not allow for modeling using a simple uni-linear fit model, we perform a more sophisticated tri-linear fit. The tri-linear fit directly assigns copy number to every sample.

### Observed read depth

Capture sequencing reads from the 1000 Genomes Project Exon Sequencing Pilot Project were downloaded, in FASTQ format, from the 1000 Genomes Project DCC site: http://1000genomes.org. The reads were mapped using the MOSAIK read mapping program, to the NCBI build 36.3 human reference genome. The resulting read alignments (in BAM format) were further processed to remove duplicate reads, and reads with low mapping quality (<20).

Gene target regions were also downloaded from the 1000 Genomes Project site. For each GSS, we determined RD as the number of distinct reads that had their first (5’) base uniquely mapped within an exon of that gene. This resulted in a matrix of RD observations (illustrated in Figure 1C left).

### Data filtering

We discarded all duplicate reads and all reads with mapping quality less than 20. We also discarded all the targets with median RD less than 30. Similarly, we discarded all the samples with median RD less than 30. In 454-sequenced data, this led to discarding almost all targets and samples; therefore we relaxed those criteria to 5 and 1, respectively. Additionally, we discarded all the genes that failed to exhibit correlation between observed RD and MRD at *r*^2^ ≥ 0.7.

### Expected read depth based on uni-linear fit and tri-linear fit

In the first attempt, we use the simple uni-linear fit; we calculate the expected read depth for normal copy number (CN=2) as the product of a gene-specific capture affinity value, α_g_, and a sample-specific measure of read coverage, the median of read depths, *MRD*_s_, across all genes for that sample:

(2) $$RD_{\mathrm{gs}}=\alpha_{g}.MRD_{s}$$

The gene-specific capture affinity (α_g_) is determined as the slope of a least-squares zero-intercept linear fit between the gene-specific read depth (*RD*_gs_) and the median read depth (*MRD*_s_) for all samples (illustrated in Figure 1B). This procedure resulted in a matrix of RD expectations (Figure 1C right).

The afore-mentioned procedure requires a single-line linear fit between *RD*_gs_ and *MRD*_s_. The quality of such a fit is evaluated by comparing *r*^2^ against a predetermined threshold (≥ 0.7 as described before). When this indicates poor quality of the single-line linear fit, we attempt to perform a tri-linear fit.

Briefly, we attempted to minimize error function

$$\text{erro}r_{g}=\sum_{s}\text{min}\left\{{\left(RD_{g,s}-\alpha_{g}MRD_{s}\right)}^{2},{\left(RD_{g,s}-\frac{\alpha_{g}}{2}MRD_{s}\right)}^{2},{\left(RD_{g,s}-0.MRD_{s}\right)}^{2}\right\},$$

where *s* iterates over samples and g indicates the gene in question. Note that the tri-linear fit directly assigns copy number to each GSS. Please see Common CNVs for more detail.

### Copy number probabilities

We used a Bayesian scheme to calculate the probability P(*CN*_*gs*_*|RD*_*gs*_ of a given copy number at a given GSS, based on the observed read depth. We only considered CN=0-9 i.e. homozygous deletion (CN=0), heterozygous deletion (CN=1), normal copy number (CN=2), and amplifications of various magnitudes (CN>2). We assigned prior probabilities P(*CN*_gs_) to each copy number based on CNV events reported in an earlier study [17] (Table 8). We assumed that, for each distinct CN, the observed RD obeys an over-dispersed Poisson distribution. Its mean value for normal copy number (CN=2) is calculated according to (Eq. 2) and for other copy numbers it is proportionally scaled. The standard deviation of the distribution includes an over-dispersion factor (ODF) in the range of 1 to 20 to account for over-dispersion (variance beyond the level of Poisson fluctuations, see Additional file 1).

Briefly, to account for over-Poisson dispersion, we used observed RD_gs_ and calculated corresponding *z*-score under an assumption of an ideal Poisson distribution at every GSS. Subsequently, we calculated a sample-specific standard deviation of that *z*-score for every sample and annotated it as sample over-dispersion factor. Similarly, we calculated a gene-specific standard deviation of *z*-score for every gene and annotated it as the gene-specific over-dispersion factor. If the assumption of an ideal Poisson distribution were true, those sample- and gene-specific standard deviations should equal 1. Subsequently, we calculated the over-dispersion factor for every GSS as a product of respective sample- and gene-specific ODFs. The ODF was then normalized and assigned to 1 if less than 1.

We used the over-dispersed Poisson distributions to calculate the data likelihoods P(*RD*_gs_|*CN*) for all considered CN values. Finally, we used Bayes’ method to estimate the posteriors for each considered CN. A CNV event is reported the posterior probability of a non-normal copy number is above a pre-defined threshold value, *h*.

### Neighboring gene deletions

A simple extension of the algorithm used neighboring gene deletion events as part of the detection method. For the purpose of our algorithm, the genes were deemed “neighboring” if they were located on the same chromosome, the segment between those genes was no longer than 3 Mbp and no gene was sequenced in between. In principle, when a gene has a deleted neighbor, we should assume a higher prior probability of a deletion in the gene in question. Since the posterior probability usually scales monotonically with the prior, for practical reasons we assumed a lower Bayesian posterior probability threshold (*h *= 0.1) to produce a preliminary list of candidate events. Events on this list for which at least one of the two immediate neighbor genes was also on the list were retained.

### Sensitivity estimation

We carried out sensitivity estimation in the SC dataset, using simple simulations. In each simulation cycle, we drew 5 genes randomly from every sample, and downscaled the observed RD for those genes by a factor of 2, to emulate heterozygous deletions. We then applied our standard detection procedure to this “spiked” dataset, and tabulated the fraction of simulated events that were detected by the algorithm.

### Common CNVs

**Figure 6:**
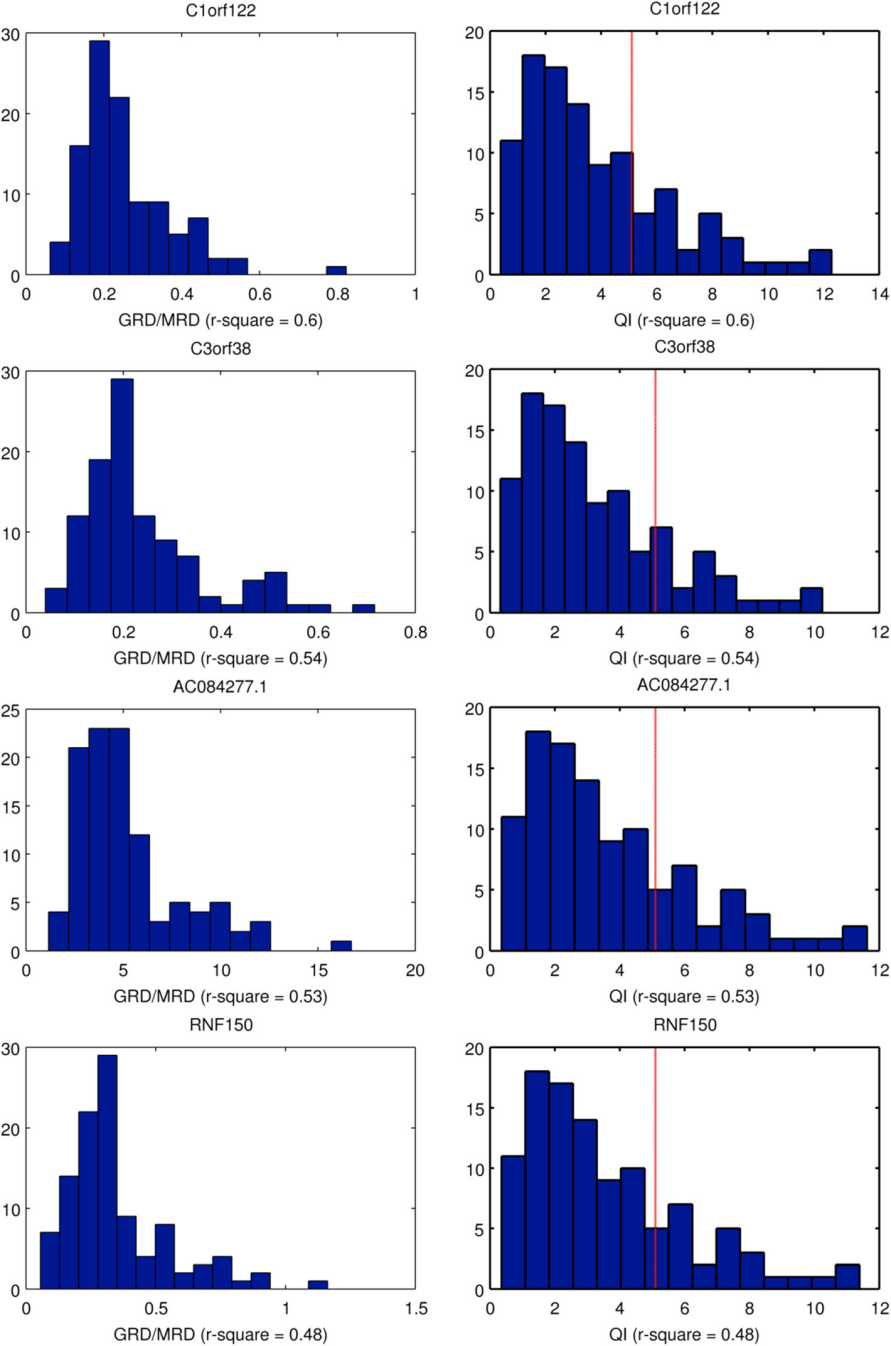
**Analysis of genes that failed simple linear fit. **Each row describes a different gene. Left panels – distribution of the ratio of RD at the GSS sites to the sample MRD. Right panels – distribution of the quality index for that gene. The non-multimodal distributions and the low quality-index values of these genes suggest that there are no common CNV events on these loci.

We evaluated all genes that failed to achieve *r*^2^ ≥ 0.7 using the linear fit model from Figure 1B. The results of that evaluation are shown in Figure 6. The last row describes result for gene RNF150 that achieved the worst *r*^2^ of 0.48. The histogram shown in the left columns demonstrates distribution of observed RD to MRD (taken as from Figure 1B), In case of a rare CNV (or lack of CNVs at all), one would expect a unimodal distribution centered around that gene affinity. For a common CNV, one additional peak corresponding to CN=1 centered around half of that gene affinity, and another peak corresponding to homozygous deletion (CN=0) around 0, should be visible. However, the data shown do not allow identifying such a pattern of either bi- or tri-modal distribution.

Additionally, the histogram of quality index calculated for that gene is presented in the right column. The low values of quality index further corroborate the conclusion that the absence of a call in that locus is due to lack of high quality data rather than due to a hypothetical common CNV event. Careful inspection of the graphs calculated for all 69 genes the failed simple linear fit reveals lack of evidence for a common CNV in any of them. Notably, in the SC dataset only 28% of GSS in genes with *r*^2^ < 0.7 were potentially detectable vs. 62% in genes with *r*^2^ ≥ 0.7.

With no common CNV present in the experimental data, we tested the sensitivity of our algorithm using simulated deletions. We used realistic gene affinities (mean and three quartiles from Figure 2B) and the empirical MRDs for 106 samples. We assumed frequency of the deleted allele among 106 samples varying from 0 to 100% in 10% increments; we allowed for random segregation, so that both homo- and heterozygous deletions were introduced. Then for each sample we calculated the expected read depth as a product of MRD and affinity; however in the samples drawn for a heterozygous deletion we used halves of the nominal affinities and in the samples drawn for a homozygous deletion, we multiplied the MRD by 0.01 to account for reads erroneously mapped into that region. Having an expected read depth *m* for each sample, we drew a random read depth using a normal distribution $N\left(m,ODF\sqrt{m}\right)\sqrt{2}$, where ODF was assumed 8.

**Figure 7:**
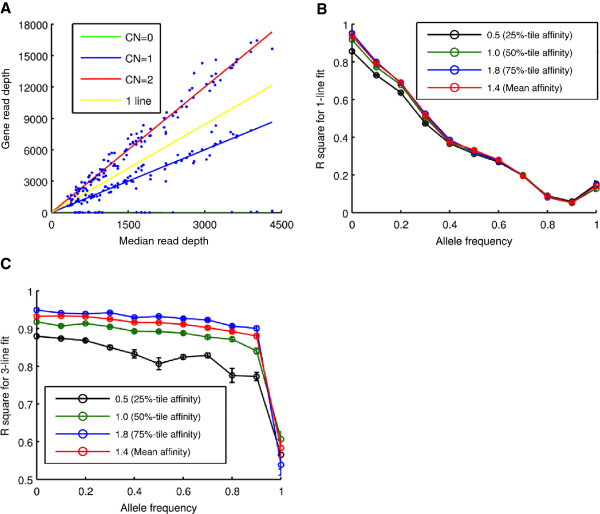
**Simulated Common CNVs. A. **If a simple linear fit fails, the gene affinity is estimated for each gene as the slope of the least-square-error tri-linear fit between MRD and RD for that gene. **B** and **C***r*^2^ values of a simple linear fit (**B**) and a tri-linear fit (**C**) as a function of the deleted allele frequency.

In Figure 7B and C we show the results of analysis performed on simulated common CNV events. Panel B shows *r*^2^ values obtained from the simple linear fit (as in Figure 1B) and panel C shows the *r*^2^ values obtained from the tri-linear fit (as in Figure 7A). The uni-linear *r*^2^ values deteriorate with the increase of the deleted allele frequency. To the contrary, the tri-linear *r*^2^ values stay relatively high over wide range of the allele frequency.

Finally, Figure 3D demonstrates that the sensitivity of the algorithm to the common CNVs remains relatively stable over wide range of the deleted allele frequency (up to 90%).

### Validation experiments

All primers were designed using primer3 (http://fokker.wi.mit.edu/primer3/) with default settings to obtain a desired PCR amplicon size between 200 bp and 250 bp. All primers were checked with BLAT (http://genome.ucsc.edu/) to avoid known SNPs that could influence primer hybridization. PCR products were run on an agarose gel to make sure they gave no additional bands besides the expected amplicon.

Primer efficiencies were determined by calculating the standard curve of a serial dilution (4 times, 10-fold) of pooled genomic DNA (Promega, Madison, WI). All experiments were performed in triplicates on the Roche LightCycler 480 platform with LightCycler 480 SYBR Green I Master (cat# 04707516001). The volume of each reaction was 20 μl with final primer concentrations of 400 nM. The PCR was performed according to the following protocol: 5 min at 95°C, 2. 45 cycles of 5 s at 95°C, 10s at 60°C, 30s at 72°C. To determine the copy number state of an event locus, we used the Delta-Delta-Ct-Method (2-ΔΔCt) for each event locus compared to a reference locus in the sample and a control pool of seven individuals (Promega, Madison, WI), respectively. This reference locus was not previously known to show any copy number variation.

Among the calls made without neighboring information, we exhaustively validated all the calls with posterior probability of 0.95 or more (4 coincided with known events [17]; we experimentally validated the remaining 18 events). Additionally, we performed qPCR validations for 4 events randomly selected from those with posterior probability between 0.65 and 0.95 (2 coincided with known events [17]; we experimentally validated the remaining 2 events).

Of the calls made with the neighboring information, we deemed 7 calls coincided with known events [17]; 7 out of 10 remaining calls were submitted for qPCR validation. For the purpose of validation, the fold change for a given gene < 0.7 was classified as a positive validation, > 0.8 as a negative validation and in the intermediate range as inconclusive.

## Abbreviations

CNV: Copy Number Variation; FDR: False Discovery Rate; RP: Read Pair; SR: Split Read; RD: Read Depth; AS: Assembly; GSS: Gene-Sample Site; WU: Washington University; SC: Wellcome Trust Sanger Institute; BI: Broad Institute; BCM: Baylor College of Medicine; MRD: Median Read Depth; CN: Copy Number; ODF: Over-Dispersion Factor; QI: Quality Index.

## Competing interests

The authors declare that they have no competing interests.

## Authors’ contributions

JW designed algorithms, performed analysis and wrote the paper. KRG designed algorithms, performed analysis, wrote the paper. CS designed algorithms and experiments. FG, AEU and MPS designed and performed the experiments. GTM designed the algorithms, wrote the paper and supervised the project. All authors read and approved the final manuscript.

## Supplementary Material

Additional file 1 Detail formula of over-dispersion factor (ODF) and quality index (QI), give the exact formula and derivation of the over-dispersion factor and quality index. Click here for file
