# Supplementary material for: Copy Number Variation detection from 1000 Genomes project exon capture sequencing data
Source: BMC Bioinformatics. 2012 Nov 17;13:305. doi: 10.1186/1471-2105-13-305 (PMC3563612; doi:10.1186/1471-2105-13-305)
Supplement: Additional file 1 — Detail formula of over-dispersion factor (ODF) and quality index (QI), give the exact formula and derivation of the over-dispersion factor and quality index. [file 1471-2105-13-305-S1.docx]

***Insight from empirical data and accounting for over-dispersion***

We performed a simulation to assess potential variability in the gene affinities on the over-dispersion. Using this data, we calculated expected read depth $RD_{\text{expected}}$ for every GSS as product of respective gene affinity and MDR. Subsequently, we calculated read depth using Poisson distribution with $RD_{\text{expected}}$ as parameter. The *z*-score calculated from that distribution followed a normal distribution $N\left(0,1\right)$, as expected for an ideal case.

Subsequently, we randomly distorted the vector of gene affinities; i.e. we drew a random number from a normal distribution $N\left(\alpha_{g},0.15\alpha_{g}\right)$ to be used instead of the exact affinity $\alpha_{g}$. With increased variability in gene affinities, the distribution becomes progressively wider; at a 15% increase in variability the results are comparable to the distribution of the empirically calculated z-score. This result indicates that as little as 15% variability in gene affinities is enough to reproduce the distribution over-dispersion observed in the experimental data.

If we knew ODF for every GSS in our data, we could correct for it, so that

$\frac{RD_{\text{observed}}-RD_{\text{expected}}}{c\sqrt{RD_{\text{expected}}}}\cong N\left(0,1\right)$,

where is the sample-gene-specific correction factor for the over-dispersed Poisson effect (over-dispersion factor, ODF).

As indicated above, ODF remains constant over a range of coverage only under assumption of mutual independence of subsequent runs. When the entire *z*-score matrix is considered, that assumption is obviously violated (i.e. RDs in different genes in a sample are correlated by sharing the same MDR and RDs in a gene in different samples are correlated by sharing gene affinity).

In the absence of a fundamental model describing interplay between gene affinities varying across genes, samples and machine runs, we developed an empirical procedure to account and correct for over-dispersion.

We estimated the over-dispersion factor for each site according to the following steps. First we calculated a “z-score” matrix ,

,

from the observed read depth matrix $\left[observed_{s,g}\right]$ and the expected read depth matrix $\left[expected_{s,g}\right]$.

Then for every row and for every column in the “z-score” matrix, we calculated their respective standard deviations. This procedure generated a column vector of row (sample-specific) standard deviations and a row vector of column (gene-specific) standard deviations. Subsequently, the over-dispersion factor matrix was calculated as

.

If any over-dispersion factor was to fall below 1, it was assigned 1 since no counting experiment of independent trials should have a variance less than that of a Poisson distribution.

Once the over-dispersion factor was calculated, we could model data likelihood using a normal distribution $N\left(expected,c\sqrt{expected}\right)$.

***Notion of quality index***

Assume that read depth in case of copy number can be approximated with normal distribution $N\left(m_{CN},\sigma_{CN}\right)$; i.e. $N\left(m_{2},\sigma_{2}\right)$ for normal copy number and $N\left(m_{1},\sigma_{1}\right)$ for a heterozygous deletion.

Then probability distribution of read depth under condition of normal copy number (2) is:

(Eq. 1)

and analogously under condition of a heterozygous deletion ():

. (Eq. 2)

The posterior probability for a particular copy number is calculated from Bayes’ theorem:

$$P\left(CN=1|data\right)=\frac{P\left(data|CN=1\right)\cdot P\left(CN=1\right)}{P\left(data\right)}$$

and

.

In order to make a call, we need the posterior probability of an event to reach certain threshold (in the paper we used *h*=0.65):

$$P\left(CN=1|data\right)\geq h$$

or, using to denote the read depth actually observed in the data, we may write the following condition which must be met by the actually observed read depth in order to make a call:

.

Here we only discuss the detection of heterozygous deletions so we assume that the posterior probability of a CN other than 1 and 2 is negligible.

The latter is equivalent to

$\frac{h}{1-h}\leq\frac{P\left(CN=1|X=x\right)}{P\left(CN=2|X=x\right)}$. (3)

By substituting ( Eq. 1) and (Eq. 2) into (Eq. 3) and rearranging the latter, we obtain

$$\frac{h}{1-h}\leq\frac{P\left(CN=1\right)}{P\left(CN=2\right)}\cdot\frac{P\left(X=x|CN=1\right)}{P\left(X=x|CN=2\right)}=$$

$$=\frac{P\left(CN=1\right)}{P\left(CN=2\right)}\cdot\frac{\sigma_{2}}{\sigma_{1}}\cdot\exp\left(-\frac{1}{2}\cdot\left(\frac{x-m_{1}}{\sigma_{1}}\right)^{2}+\frac{1}{2}\cdot\left(\frac{x-m_{2}}{\sigma_{2}}\right)^{2}\right)$$

and subsequently

$$\frac{h}{1-h}\cdot\frac{P\left(CN=2\right)}{P\left(CN=1\right)}\cdot\frac{\sigma_{1}}{\sigma_{2}}\leq\exp\left(-\frac{1}{2}\cdot\left(\frac{x-m_{1}}{\sigma_{1}}\right)^{2}+\frac{1}{2}\cdot\left(\frac{x-m_{2}}{\sigma_{2}}\right)^{2}\right)$$

$$2\ln\left(\frac{h}{1-h}\cdot\frac{P\left(CN=2\right)}{P\left(CN=1\right)}\cdot\frac{\sigma_{1}}{\sigma_{2}}\right)\leq-\left(\frac{x-m_{1}}{\sigma_{1}}\right)^{2}+\left(\frac{x-m_{2}}{\sigma_{2}}\right)^{2}$$

$\begin{matrix} 2\ln\left(\frac{h}{1-h}\cdot\frac{P\left(CN=2\right)}{P\left(CN=1\right)}\cdot\frac{\sigma_{1}}{\sigma_{2}}\right) \\ \leq x^{2}\left(\frac{-1}{\sigma_{1}^{2}}+\frac{1}{\sigma_{2}^{2}}\right)+x\left(\frac{2m_{1}}{\sigma_{1}^{2}}-\frac{2m_{2}}{\sigma_{2}^{2}}\right)+\left(\frac{-m_{1}^{2}}{\sigma_{1}^{2}}+\frac{m_{2}^{2}}{\sigma_{2}^{2}}\right) \end{matrix}$.

Finally, that yields the following condition on the actually observed read depth necessary to make a call:

$\begin{matrix} 2\ln\left(\frac{h}{1-h}\cdot\frac{P\left(CN=2\right)}{P\left(CN=1\right)}\cdot\frac{\sigma_{1}}{\sigma_{2}}\right) \\ \leq x^{2}\left(\frac{-1}{\sigma_{1}^{2}}+\frac{1}{\sigma_{2}^{2}}\right)+x\left(\frac{2m_{1}}{\sigma_{1}^{2}}-\frac{2m_{2}}{\sigma_{2}^{2}}\right)+\left(\frac{-m_{1}^{2}}{\sigma_{1}^{2}}+\frac{m_{2}^{2}}{\sigma_{2}^{2}}\right) \end{matrix}$. (Eq. 4)

Assuming the distributions approximate Poisson distribution with some over-dispersion factor, i.e. by substituting

$\sigma_{2}=\kappa\sqrt{m_{2}}$ (Eq. 5)

$\sigma_{1}=\kappa\sqrt{m_{1}}$ (Eq. 6)

into (Eq. 4), we obtain

$$2\ln\left(\frac{h}{1-h}\cdot\frac{P\left(CN=2\right)}{P\left(CN=1\right)}\cdot\frac{\kappa\sqrt{m_{1}}}{\kappa\sqrt{m_{2}}}\right)$$

$$\kappa^{2}2\ln\left(\frac{h}{1-h}\cdot\frac{P\left(CN=2\right)}{P\left(CN=1\right)}\cdot\frac{\sqrt{m_{1}}}{\sqrt{m_{2}}}\right)$$

$$\leq x^{2}\left(\frac{-1}{m_{1}}+\frac{1}{m_{2}}\right)+x\left(\frac{2m_{1}}{m_{1}}-\frac{2m_{2}}{m_{2}}\right)+\left(\frac{-m_{1}^{2}}{m_{1}}+\frac{m_{2}^{2}}{m_{2}}\right)$$

and given that

we obtain

. (Eq. 7)

We may therefore conclude that if the right hand side of (Eq. 7) is negative, then there is no read depth such that a call would be made. Comparing RHS to 0 gives:

. (Eq. 8)

However, the left hand side of (Eq. 8) equals, by our definition, the quality index

, (Eq. 9)

so that the right hand side of (Eq. 8) defines the minimum value of quality index necessary to make a call even possible:

$q_{\min}=2\sqrt{\ln\left(\frac{h}{1-h}\cdot\frac{P\left(CN=2\right)}{P\left(CN=1\right)}\cdot\sqrt{\frac{1}{2}}\right)}$. (Eq. 10)

We may now ask the following question: assuming that copy number is 1, what is the probability of observing read depth greater than detection threshold?

If the right hand side of (Eq. 7) is positive (i.e. detection is possible), then by taking square root of (Eq. 7), we obtain the following condition that read depth must satisfy in order to make a call:

,

which, using symbols from (Eq. 10), can be written as

$x\leq\sqrt{-m_{2}\kappa^{2}\cdot\frac{q_{\min}^{2}}{2}+\frac{m_{2}^{2}}{2}}$. (Eq. 11)

Obviously, under assumption of copy number 1, (Eq. 2) will apply. Therefore, probability of obtaining satisfy (Eq. 11) will be

$DE=\mathrm{norm}\_\mathrm{cdf}\left(\frac{\sqrt{-m_{2}\kappa^{2}\cdot\frac{q_{\min}^{2}}{2}+\frac{m_{2}^{2}}{2}}-m_{1}}{\sigma_{1}}\right)$, (Eq. 12)

where $\mathrm{norm}\_\mathrm{cdf}$ is cumulative distribution function of the normal distribution. By substituting (Eq. 5) and (Eq. 6), and transforming (Eq. 12), we obtain

$$DE=\mathrm{norm}\_\mathrm{cdf}\left(\frac{\sqrt{-m_{2}\kappa^{2}\cdot\frac{q_{\min}^{2}}{2}+\frac{m_{2}^{2}}{2}}-\frac{m_{2}}{2}}{\kappa\sqrt{\frac{m_{2}}{2}}}\right)$$

$$=\mathrm{norm}\_\mathrm{cdf}\left(\frac{\sqrt{\frac{1}{2}}\sqrt{1-\frac{q_{\min}^{2}}{q^{2}}}-\frac{1}{2}}{\frac{1}{q}\sqrt{\frac{1}{2}}}\right)$$

$$=\mathrm{norm}\_\mathrm{cdf}\left(q\cdot\left(\sqrt{1-\frac{q_{\min}^{2}}{q^{2}}}-\sqrt{\frac{1}{2}}\right)\right)$$

$=\mathrm{norm}\_\mathrm{cdf}\left(\sqrt{q^{2}-q_{\min}^{2}}-q\sqrt{\frac{1}{2}}\right)$. (Eq. 13)

Note that detection efficiency $DE$approximated in that manner depends directly only on and .

From (Eq. 13) we can conclude that 50% detection efficiency would be achieved when

$\sqrt{q^{2}-q_{\min}^{2}}-q\sqrt{\frac{1}{2}}=0$,

i.e.

$q=q_{\min}\sqrt{2}$.

At *q*_min_=5.5 (as calculated in our manuscript), one needs .

The property of quality index can be used to specify the requirements on sample MRD and base coverage.

In the actual data collected from sample NA12234, we saw 622 (72%) out of 862 genes with quality index at least 5.4 (i.e. being potentially *detectable*). However, only 382 (44%) genes have the quality index of 7.8 or more, allowing for 50% of detection efficiency. Two-fold increase in coverage would make 803 (93%) genes detectable and 602 (70%) genes having 50% of detection efficiency. Three-fold increase in coverage would yield make 845 (98%) genes detectable and 734 (85%) genes having 50% of detection efficiency.

Analogously to (Eq. 12), we may calculate false positive rate (probability of making a false call under assumption that the true copy number is 2):

$FPR=\mathrm{norm}\_\mathrm{cdf}\left(\frac{\sqrt{-m_{2}\kappa^{2}\cdot\frac{q_{\min}^{2}}{2}+\frac{m_{2}^{2}}{2}}-m_{2}}{\sigma_{2}}\right)$, (Eq. 14)

which transforms into

$$FPR=\mathrm{norm}\_\mathrm{cdf}\left(\frac{\sqrt{-m_{2}\kappa^{2}\cdot\frac{q_{\min}^{2}}{2}+\frac{m_{2}^{2}}{2}}-m_{2}}{\kappa\sqrt{m_{2}}}\right)=$$

$$=\mathrm{norm}\_\mathrm{cdf}\left(\frac{\sqrt{-\frac{\kappa^{2}}{m_{2}}\cdot\frac{q_{\min}^{2}}{2}+\frac{1}{2}}-1}{\kappa\sqrt{\frac{1}{m_{2}}}}\right)=$$

$$=\mathrm{norm}\_\mathrm{cdf}\left(\frac{\sqrt{\frac{1}{2}}\sqrt{-\frac{q_{\min}^{2}}{q^{2}}+1}-1}{\frac{1}{q}}\right)=$$

$=\mathrm{norm}\_\mathrm{cdf}\left(q\sqrt{\frac{1}{2}}\sqrt{-\frac{q_{\min}^{2}}{q^{2}}+1}-q\right)$,

so that finally

$FPR=\mathrm{norm}\_\mathrm{cdf}\left(\sqrt{\frac{1}{2}}\sqrt{q^{2}-q_{\min}^{2}}-q\right)$. (Eq. 15)
